# Meningitis Caused by* Salmonella Newport* in a Five-Year-Old Child

**DOI:** 10.1155/2016/2145805

**Published:** 2016-12-12

**Authors:** Ana De Malet, Sheila Ingerto, Israel Gañán

**Affiliations:** ^1^Microbiology Department, Hospital Comarcal Valdeorras, Avenida Conde de Fenosa, No. 50, CP 32300, O Barco, Ourense, Spain; ^2^Pediatrics Department, Hospital Comarcal Valdeorras, Avenida Conde de Fenosa, No. 50, CP 32300, O Barco, Ourense, Spain; ^3^Immunology Department, Hospital Universitario Ramón y Cajal, Ctra Colmenar Viejo, Km 9,100, CP 28034, Madrid, Spain

## Abstract

*Salmonella Newport* is a Gram-negative bacillus belonging to the Enterobacteria family and the nontyphi Salmonella (NTS), usually related to gastroenteritis. Main difference between NTS and* Salmonella typhi* is that the last one evolves to an invasive disease easier than NTS. These can progress to bacteremias in around 5% of cases and secondary focuses can appear occasionally, as in meningitis. An infection of the central nervous system is uncommon, considering its incidence in 0.6–8% of the cases; most of them are described in developing countries and mainly in childhood, especially neonates. Bacterial meningitis by NTS mostly affects immunosuppressed people in Europe. Prognosis is adverse, with a 50% mortality rate, mainly due to complications of infection: hydrocephalus, ventriculitis, abscesses, subdural empyema, or stroke. Choice antibiotic treatments are cefotaxime, ceftriaxone, or ceftazidime. The aim of this paper is to present a case of meningitis caused by* Salmonella Newport* diagnosed in a five-year-old girl living in a rural area of the province of Ourense (Spain), with favorable evolution and without neurological disorders.

## 1. Introduction

Meningitis by* Salmonella *sp. is a rare infection, considered a complication of salmonellosis that affects mostly children and immunosuppressed patients. One rare association in immunosuppressed patients with recurrent bacterial infections is the deficiency of Mannose-binding lectin (MBL), where the antibiotic and steroid therapy is not effective [1]. It has very rapid evolution and clinical manifestations indistinguishable from any other bacterial meningitis. It is traditionally associated with a high incidence of complications, neurological disorders, high mortality, and a high percentage of relapses. These situations have been reported particularly in cases where* Salmonella typhi *was isolated in children with early age [2]. Third-generation cephalosporin (3-4 weeks) is the treatment of choice.

## 2. Case Presentation

The authors report a case of a five-year-old child who came to the Emergency Department of Comarcal Valdeorras Hospital (Ourense, Galicia, Spain) with a less than 24 hours' clinical presentation with high fever, vomiting, and drowsiness. Medical history revealed that the patient had had chickenpox the previous week; she was properly vaccinated, having no previous serious infections.

The mother reported no pets or contact with farm animals. Upon admission, patient showed normal vital sign ranges (AP: 110/50 mmHg, oxygen saturation 97%) and axillary temperature of 39°C with poor response to antipyretics. Physical examination revealed general malaise, skin pallor, neck stiffness, neurological affectation with decreased Glasgow (14-15), and positive meningeal signs.

Blood count, complete biochemical analysis, acute-phase proteins determination, blood culture inoculated in a pediatric bottle BactAlert® BioMérieux system, and lumbar puncture for cellular and microbiological study were made. Laboratory findings showed leukopenia with neutropenia and acute-phase reactants, C-reactive protein (CRP), and procalcitonin level slightly elevated (see Table 1: analysis 1). Platelets, electrolytes, glucose, creatinine, transaminases, and bilirubin were within normal ranges. Complement studies including CH50, AH50, C3, and C4 levels were normal. Cerebrospinal fluid (CSF) cell count was reported with 1600 cells/*μ*L with predominance of polymorphonuclear leukocytes (96%), glucose 50 mg/dL, and proteins 80 mg/dL. No microorganisms were observed in direct Gram's stain. CSF was plated onto chocolate blood agar plates incubated at 37°C in a 10% CO_2_ atmosphere and in MacConkey agar plates and thioglycolate broth incubated at 37°C. Viral study was not performed because of the shortage of the CSF sample.

An analysis was performed at 12 hours of admission, showing leukocytosis with neutrophilic and acute-phase proteins elevated (see Table 1: analysis 2).

After 24 hours of CSF incubation, turbidity was observed in thioglycolate broth. A single large, gray, and smooth colony was isolated in chocolate blood agar and a large and lactose negative colony in MacConkey agar, both of them compatible with Enterobacteria. A colony of Gram-negative bacilli was obtained in both Gram stain and thioglycolate broth. The identification was made using the automated system Vitek® BioMérieux with GN card as* Salmonella *sp.; it was confirmed by conventional biochemical tests, while in vitro susceptibility testing was performed using the same system and 243 cards, being interpreted according to the criteria defined by Clinical and Laboratory Standards Institute for Enterobacteriaceae [3].* Salmonella *sp. was susceptible to ceftriaxone, cefotaxime, amoxicillin-clavulanate, imipenem, ciprofloxacin, azithromycin, and trimethoprim-sulfamethoxazole. The blood culture bottle was positive after 3 days of incubation in an aerobic atmosphere and 37°C, so that* Salmonella* sp. with the same antibiogram compared to the organism isolated from CSF was cultured. Knowing those microbiological findings, a fecal culture was performed in patient and her close relatives, parents, and brother, but in all cases normal microbiota was observed. Also, there was no evidence of any other case of salmonellosis in the area in that time, ruling its association with a food outbreak.

The strain was sent to the Spanish National Microbiology Center at the Carlos III Institute (Majadahonda, Madrid), where it was typified as* Salmonella Newport*.

With the analytical data of CSF and the suspicion of bacterial meningitis, a treatment with intravenous cefotaxime (200 mg/kg/day) was begun. After the diagnosis of meningitis by* Salmonella *sp. was confirmed, treatment was maintained for 3 weeks associated with intravenous dexamethasone (0.15 mg/kg) every 6 hours for 48 hours. After removing treatment, the patient remained afebrile with good general condition, without neurological disorders, with good oral intake, without vomiting, and with normal bowel movements. After remaining stable after 3 weeks of hospitalization, normal blood count, CRP: 0.3 mg/dL, and procalcitonin: 0.22 ng/mL, the patient was discharged with follow-up in pediatric outpatient center.

During hospitalization, the use of a computerized tomography (CT) as imaging test to discard intracranial complications was proposed, but given her correct and rapid clinical course and technical problems in the CT it was decided not to do it. On the other hand, any study of latent immunosuppression or genetically acquired susceptibility to acute bacterial infections was carried out by the good evolution of infection with treatment and the absence of multiple infections in the clinical history of the patient or close relatives.

At 6 months of the infection, the patient had a favorable evolution without physical or neurological disorders.

## 3. Discussion

*Salmonella *sp. is a facultative anaerobic Gram-negative bacillus, mobile, hydrogen sulphide producing bacteria belonging to the family Enterobacteriaceae.

Classically three primary pathogenic species were distinguished:* S. typhi*,* S. choleraesuis, *and* S. enteritidis*, the two last belonging to the NTS group. However, according to the Kauffman-White-Le Mino serotyping [4], they are classified into more than 2000 serotypes based on flagellar antigens H (protein) and somatic antigens O (polysaccharide fraction of bacillary lipopolysaccharide). Additionally,* S. typhi *has a virulence antigen (Vi) [4].

NTS is usually related to self-limited gastroenteritis, evolving to bacteremia from 1 to 5.7% of cases [5] and rarely spreading through the blood to other locations. It may exceptionally appear as secondary outbreaks of meningeal infection [6, 7].

The first known case of* Salmonella meningitis* was described in 1907 by Ghon et al. [8]. After that, relevant publications about this subject have been scarce, limited to isolated cases or series with a low number of patients, which shows the infrequency of the pathology. Its incidence is estimated between 0.8 and 6% of cases of bacterial meningitis [5], although there are differences between developed and developing countries: from 0.1% in Britain to 12% in Malawi. It mainly affects children under 5 years, especially infants (6%) and neonates (16%) [5, 8].

Isolating cases have been reported from Spain; most of them refer to immunocompromised adults [5, 7, 9, 10]. Immunosuppression by some kind of disease or immunological immaturity stands as a risk factor, related to a decreasing capacity to opsonize bacteria in the affected patients. Mannose-binding lectin (MBL) is an important component of the innate immune system. It binds to the arrays of sugars commonly presented by microorganisms and activates the complement system independently of antibody. MBL functions as an opsonin which enhances the sequential immune process such as phagocytosis. A study reported an inhibitory effect of MBL on the motility of Salmonella, which occurs by affecting the energy source required for motility and the signaling pathway of chemotaxis [11].

Deficiency of MBL or absence of functional MBL activity can be the cause of recurrent infections. Three genetic mutations in the MBL gene are known to result in low levels of circulating MBL [12]. In these cases antibiotic and associated steroid therapy had been described of little benefit [12]. Our patient shows a good response of treatment and no history of recurrent infections was found in the patient or close relatives. For this reason, any study was proposed about it.

Traumatic births and increased permeability of the hematoencephalic barrier also are considered risk factors in newborns [8]. Prognosis is adverse, with 50% mortality mainly due to complications of infection: hydrocephalus, ventriculitis, abscesses, subdural empyema, or stroke [2].

*Salmonella* sp. serotypes most frequently associated with meningitis are* S. typhimurium* (75–88%), followed by* S. enteritidis* (8–16%) and less frequently* S. typhi* (1–4%) [2]. In this report, we present a case in which* Salmonella Newport*, belonging to the* S. enteritidis* group C serotype, was isolated.

The most common mechanism of transmission is ingestion of contaminated food although other rarer routes have been described as transplacental, through breast milk or by contact with reptiles carriers of this organism [8]. Gastrointestinal syndrome as evidence of infection has only been described in 22–40% of cases in developing countries [12]. In the case described neither did the patient refer to gastrointestinal syndrome nor could the source of infection be documented because the bacteria were not isolated in the stool culture. We assume that this is a false negative by collecting the sample after that the antibiotic treatment was initiated. The authors want to emphasize that the initial immunosuppression probably due to chicken pox episode could have stimulated the spread of the organism through the blood to the central nervous system.

Treatment of* Salmonella *sp. meningitis needs drugs with good spread to cross the hematoencephalic barrier, but also because* Salmonella *sp. are a facultative intracellular bacteria and the wrong penetration of the antimicrobial into cells promotes the spread of the organism and the emergence of antibiotic resistance. Due to its bioavailability characteristics, third-generation cephalosporins such as cefotaxime, ceftriaxone, or ceftazidime are considered the treatment of choice, with a minimum duration of 3-4 weeks to prevent relapses, which have been described in 60% of cases [13]. Use of fluoroquinolones has also been documented, especially in cases of treatment failure or relapse after initial treatment with cephalosporins, both associated with them or as monotherapy [14, 15]. This is because although the amount of drug reaching the CSF is three times lower than in serum, however, it is still greater than the minimum inhibitory concentration (MIC) of* Salmonella* sp. strains sensitive to fluoroquinolones. Carbapenems, mainly meropenem, have also been described as therapeutic alternative [15, 16]. The association of steroids to avoid side effects is controversial by probable underlying immunosuppression associated with invasive salmonellosis [17].

Finally, meningitis by* Salmonella enteritidis *is a rare infection associated with high mortality and neurological complications, being essential its early diagnosis and treatment.

## Figures and Tables

**Table 1:** Blood count and biochemical analysis in peripheral blood.

	Analysis 1	Analysis 2	Units	Range
Hematocrit	34.9	37	%	36.0–47.0
Leukocytes	3.2 · 10^3^	16 · 10^3^	cell/*μ*L	4 · 10^3^–11 · 10^3^
Neutrophils	1.3 · 10^3^	11 · 10^3^	cell/*μ*L	1.70–7.50
	42	70	%	42–74
Lymphocytes	1.5 · 10^3^	3.5 · 10^3^	cell/*μ*L	1.00–3.50
	48	22	%	16–45
CRP	4	10.4	mg/dL	0.0–5
Procalcitonin	2	7	ng/mL	0.0–0.5
Glucose	90	80	mg/dL	70–110
Creatinine	0.47	0.49	mg/dL	0.30–1.10
Bilirubin	0.39	0.36	mg/dL	0.20–1.2
AST/GOT	25	30	U/L	4–50
ALT/GPT	15	17	U/L	5–40
Na+	138	140	mM/L	135–148
K+	3.8	3.5	mM/L	3.5–5.2
